# Protective Effects of *Galium verum L.* Extract against Cardiac Ischemia/Reperfusion Injury in Spontaneously Hypertensive Rats

**DOI:** 10.1155/2019/4235405

**Published:** 2019-02-04

**Authors:** Jovana Bradic, Vladimir Zivkovic, Ivan Srejovic, Vladimir Jakovljevic, Anica Petkovic, Tamara Nikolic Turnic, Jovana Jeremic, Nevena Jeremic, Slobodanka Mitrovic, Tanja Sobot, Nenad Ponorac, Marko Ravic, Marina Tomovic

**Affiliations:** ^1^University of Kragujevac, Faculty of Medical Sciences, Department of Pharmacy, Svetozara Markovica 69, 34 000 Kragujevac, Serbia; ^2^University of Kragujevac, Faculty of Medical Sciences, Department of Physiology, Svetozara Markovica 69, 34 000 Kragujevac, Serbia; ^3^University IM Sechenov, 1st Moscow State Medical, Department of Human Pathology, Trubetskaya Street 8, 119991 Moscow, Russia; ^4^University of Kragujevac, Faculty of Medical Sciences, Department of Pathology, Svetozara Markovica 69, 34 000 Kragujevac, Serbia; ^5^University of Banja Luka, Faculty of Medicine, Department of Physiology, Save Mrkalja 14, 78000 Banja Luka, Bosnia and Herzegovina

## Abstract

*Galium verum L.* (*G*. *verum*, *lady's bedstraw*) is a perennial herbaceous plant, belonging to the Rubiaceae family. It has been widely used throughout history due to multiple therapeutic properties. However, the effects of this plant species on functional recovery of the heart after ischemia have still not been fully clarified. Therefore, the aim of our study was to examine the effects of methanol extract of *G. verum* on myocardial ischemia/reperfusion (I/R) injury in spontaneously hypertensive rats (SHR), with a special emphasis on the role of oxidative stress. Rats involved in the research were divided randomly into two groups: control (spontaneously hypertensive rats (SHR)) and *G. verum* group, including SHR rats treated with the *G. verum* extract (500 mg/kg body weight per os) for 4 weeks. At the end of the treatment, *in vivo* cardiac function was assessed by echocardiography. Rats were sacrificed and blood samples were taken for spectrophotometric determination of systemic redox state. Hearts from all rats were isolated and retrogradely perfused according to the *Langendorff* technique. After a stabilization period, hearts were subjected to 20-minute ischemia, followed by 30-minute reperfusion. Levels of prooxidants were spectrophotometrically measured in coronary venous effluent, while antioxidant enzymes activity was assessed in heart tissue. Cell morphology was evaluated by hematoxylin and eosin (HE) staining. 4-week treatment with *G. verum* extract alleviated left ventricular hypertrophy and considerably improved *in vivo* cardiac function. Furthermore, *G. verum* extract preserved cardiac contractility, systolic function, and coronary vasodilatory response after ischemia. Moreover, it alleviated I/R-induced structural damage of the heart. Additionally, *G. verum* extract led to a drop in the generation of most of the measured prooxidants, thus mitigating cardiac oxidative damage. Promising potential of *G. verum* in the present study may be a basis for further researches which would fully clarify the mechanisms through which this plant species triggers cardioprotection.

## 1. Introduction

Acute myocardial infarction (AMI) is one of the most common causes of disability and mortality worldwide [1]. Although timely restoration of blood flow to an ischemic heart is essential for limiting the infarct size, it can paradoxically exacerbate tissue damage. This phenomenon is known as ischemia/reperfusion (I/R) injury and occurs in several forms such as reperfusion-induced arrhythmias, myocardial stunning, microvascular obstruction, and reperfusion-induced cardiomyocyte death [2]. The exact mechanisms underlying progression of I/R injury have been under intense investigation for more than four decades with the aim to open the way to novel effective strategies for myocardial salvage [3]. Factors that have been proven to mostly contribute to myocardial dysfunction are increased generation of prooxidants, pronounced inflammatory response, and intracellular and mitochondrial Ca^2+^ overload [3, 4]. Presence of hypertension, as one of the most common risk factors for cardiovascular diseases, may worsen I/R outcome due to high myocardial load/mechanical stress [5].

Ischemic preconditioning is a successful maneuver for reaching cardioprotection by exposing the heart to brief episodes of ischemia and reperfusion prior to prolonged ischemia. Due to its limited clinical application, recent researches have focused its attention on agents that might be pharmacological mimetics of ischemic preconditioning [4]. Growing evidence suggest benefits of dietary intake of medicinal plants rich in polyphenols in lowering the incidence of coronary diseases [6, 7]. Polyphenols are biologically active constituents found in vegetables, fruits, wines, etc., with great potential to attenuate the deleterious impact of I/R via antioxidant and anti-inflammatory activities and preservation of nitric oxide [7].

*Galium verum* (*G*. *verum*, *lady's bedstraw*) is a perennial herbaceous plant, belonging to the Rubiaceae family and has been widely recognized throughout history due to its multiple therapeutic properties [8]. It has been used as a sedative, an anticancer agent, in the treatment of gout and epilepsy; nevertheless, effects of this plant species on cardiac function have not been clarified yet [9]. Phytochemical investigations revealed the presence of iridoid glycosides, phenolic compounds (such as chlorogenic and caffeic acids, hyperoside), anthraquinones, and triterpenes in *G. verum* extracts [8, 9]. Therefore, we hypothesized that *G. verum* extract might be involved in the alleviation of harmful effects of I/R injury on functional and morphological properties of the heart.

Regarding above-mentioned data the aim of our study was to assess the effects of methanol extract of *G. verum* on myocardial I/R injury in spontaneously hypertensive rats (SHR), with a special emphasis on the role of oxidative stress.

## 2. Materials and Methods

### 2.1. Ethical Approval

This investigation was carried out in the laboratory for cardiovascular physiology of the Faculty of Medical Sciences, University of Kragujevac, Serbia. The study protocol was approved by the Ethical Committee for the welfare of experimental animals of the Faculty of Medical Sciences, University of Kragujevac, Serbia. All experiments were performed according to the EU Directive for the welfare of laboratory animals (86/609/EEC) and the principles of Good Laboratory Practice (GLP).

### 2.2. Plant Material and Extract Preparation

The whole plant *G. verum* was collected on July 5th, 2017 in the village *Dobroselica* on the southern cliff of *Mt. Zlatibor (GPS coordinates: 43°42*′*59.99* ^″^ *N and 19° 41*′*59.99* ^″^ *E).* Identification and classification of the plant material were performed at the Department of Biology, Faculty of Natural Sciences, University of Kragujevac and at the Institute of Botany and Botanical garden “Jevremovac”, University of Belgrade. A voucher specimen (number 17417) was kept in a herbarium of the Institute of Botany and Botanical garden “Jevremovac”. The collected material was dried under the shade and powdered (sieve 0.75). The methanol extract was prepared to extract 100 g of the aerial part of the plant with 500 ml of methanol by heat reflux extraction [10]. The mixture was filtered through a filter paper (Whatman, No. 1), and the dry extract was obtained after removing the solvent by evaporation under reduced pressure (RV05 basic IKA, Germany). Afterwards, the residue (16.03 g) was stored in a dark glass bottle at +4°C until use. Once daily just before administration to experimental animals, extract was dissolved in the tap water.

### 2.3. Animals

Twenty spontaneously hypertensive rats (males, eight weeks old, body weight 150 ± 30 g) were included in the study. They consumed commercial rat food (20% protein rat food, Veterinary Institute Subotica, Serbia) *ad libitum* and were housed at a temperature of 22 ± 2°C, with 12 hours of automatic illumination daily. The rats were divided into two groups as follows:

*(1) Control Group*. Rats who drank only tap water

*(2) G. verum Group*. Rats who drank tap water containing 500 mg/kg of methanol extract of *G. verum*

In order to provide a precise amount of drug for every animal, rats were kept in separate cages (one animal per cage). Considering that animals drink approximately 50 ml of water per day, we dissolved individual dose of extract adjusted to body weight (500 mg/kg) in this volume of water. The same procedure was repeated every day of the experimental protocol.

### 2.4. Assessment of In Vivo Cardiac Function

Transthoracic echocardiograms were performed after accomplishing drinking protocol. A mixture of ketamine 50 mg/kg (100 mg/mL; *Ketaset*, *Fort Dodge*, *IA*) and xylazine 10 mg/kg (100 mg/mL; *AnaSed*, *Lloyd Laboratories*, *Shenandoah*, *IA*) intraperitoneally was used as anesthesia. Echocardiograms were performed using a Hewlett-Packard Sonos 5500 (Andover, Massachusetts) sector scanner equipped with a 15.0 MHz phased array transducer as previously described [11]. From the parasternal long-axis view in a 2-dimensional mode, M-mode cursor was positioned perpendicularly to the interventricular septum and posterior wall of the left ventricle (LV) at the level of the papillary muscles and M-mode images were obtained. Interventricular septal wall thickness at end diastole (IVSd), LV internal dimension at end diastole (LVIDd), LV posterior wall thickness at end diastole (LVPWd), interventricular septal wall thickness at end systole (IVSs), LV internal diameter at end systole (LVIDs), and LV posterior wall thickness at end systole (LVPWs) were recorded with M-mode. Fractional shortening percentage (FS%) was calculated from the M-mode LV diameters using the equation: [(LVEDd − LVESd)/LVEDd] × 100%.

### 2.5. Preparation of Isolated Rat Hearts

Following 4 weeks of *G. verum* extract treatment, after short-term narcosis induced by intraperitoneal application of ketamine (10 mg/kg) and xylazine (5 mg/kg) and premedication with heparin as an anticoagulant, animals were sacrificed by decapitation. Then the chest was opened via midline thoracotomy, the hearts were immediately removed and immersed in cold saline, and the aortas were cannulated and retrogradely perfused under a constant perfusion pressure (CPP) of 70 cmH_2_O according to the *Langendorff* technique. The composition of Krebs–Henseleit buffer used for retrograde perfusion was as follows (mmol/L): NaCl 118, KCl 4.7, CaCl_2_ × 2H_2_O2.5, MgSO_4_ × 7H_2_O 1.7, NaHCO_3_ 25, KH_2_PO_4_ 1.2, glucose 11, pyruvate 2, equilibrated with 95% O_2_ plus 5% CO_2_, and warmed to 37°C (pH 7.4).

### 2.6. Assessment of Ex Vivo Cardiac Function

After placing the sensor (transducer BS473-0184, Experimetria Ltd., Budapest, Hungary) in the left ventricle, the following parameters of myocardial function have been measured during stabilization and during reperfusion: maximum rate of pressure development in the left ventricle (dp/dt max), minimum rate of pressure development in the left ventricle (dp/dt min), systolic left ventricular pressure (SLVP), diastolic left ventricular pressure (DLVP), and heart rate (HR). Coronary flow (CF) was measured flowmetrically.

At first, the hearts underwent a 30-minute perfusion at CPP of 70 cmH_2_O in order to achieve a stable rhythm. After a stabilization period, the hearts were exposed to global ischemia (perfusion was totally stopped) for 20 minutes, followed by 30 minutes of reperfusion. All cardiodynamic parameters and coronary flow were measured in intervals of 5 minutes during the period of reperfusion (30 minutes) (RP1-RP7).

### 2.7. Biochemical Analysis

After sacrificing, blood samples for biochemical analysis were collected from a jugular vein in order to test the systemic redox state. After centrifugation of heparinised venous blood, plasma and erythrocytes were separated. In plasma, the following prooxidants were determined: the levels of superoxide anion radical (O_2_^−^), nitrites (NO_2_^−^), hydrogen peroxide (H_2_O_2_), and index of lipid peroxidation (measured as thiobarbituric acid reactive substances (TBARS)). Parameters of the antioxidative defense system such as activities of superoxide dismutase (SOD) and catalase (CAT) and level of reduced glutathione (GSH) were determined in erythrocyte samples. Cardiac oxidative stress markers were determined in coronary venous effluent and heart tissue. After heart stabilization and in the intervals of 5 minutes during the period of reperfusion, coronary venous effluent was collected and used for spectrophotometric determination of levels of TBARS, O_2_^−^, H_2_O_2_, and NO_2_^−^. Analyses of the prooxidants were performed using the same methods as when analyzing plasma samples and coronary venous effluent except for NO_2_^−^ where the protocol differs. The activity of antioxidant enzymes such as CAT and SOD was determined in heart tissue.

#### 2.7.1. Superoxide Anion Radical Determination (O_2_^−^)

The level of superoxide anion radical (O_2_^−^) was determined using nitro blue tetrazolium (NBT) reaction in TRIS-buffer combined with plasma sample or coronary venous effluent. The measurement was performed at a wavelength of 530 nm. Krebs–Henseleit solvent was used as the blank control for O_2_^−^ determination in coronary venous effluent, while distilled water for plasma samples [12].

#### 2.7.2. Hydrogen Peroxide Determination (H_2_O_2_)

The protocol for measurement of hydrogen peroxide (H_2_O_2_) is based on oxidation of phenol red in the presence of horseradish peroxidase. 200 *μ*l sample with 800 *μ*l PRS (phenol red solution) and 10 *μ*l POD (horseradish peroxidase) were combined (1 : 20). The level of H_2_O_2_ was measured at 610 nm. Krebs–Henseleit solvent was used as the blank control for the determination of this parameter in coronary venous effluent, while distilled water was used for plasma samples [13].

#### 2.7.3. Nitrite Determination (NO_2_^−^)

Nitric oxide (NO) decomposes rapidly to form stable metabolite nitrite/nitrate products. Nitrite (NO_2_^−^) was determined as an index of nitric oxide production with Griess reagent. For NO_2_^−^ determination in plasma 0.1 ml 3 N PCA (perchloride acid), 0.4 ml 20 mM ethylenediaminetetraacetic acid (EDTA) and 0.2 ml plasma were put on ice for 15 min, then centrifuged for 15 min at 6000 rpm. After pouring off the supernatant, 220 *μ*l K_2_CO_3_ was added. Nitrites were measured at 550 nm. Distilled water was used as a blank probe.

In order to determine NO_2_^−^ level in coronary venous effluent, 0.5 ml of the perfusate was precipitated with 200 *μ*l of 30% sulfosalicylic acid, mixed for 30 min, and centrifuged at 3000 x g. Equal volumes of the supernatant and Griess reagent were mixed and stabilized for 10 min in the dark, and then the sample was measured spectrophotometrically at a wavelength of 543 nm. The nitrite concentrations were determined using sodium nitrite as the standard [14].

#### 2.7.4. Determination of the Index of Lipid Peroxidation Measured as TBARS

The degree of lipid peroxidation in the sample (plasma and coronary venous effluent) was estimated by measuring of TBARS using 1% TBA (thiobarbituric acid) in 0.05 NaOH, incubated with sample at 100°C for 15 min, and read at 530 nm. TBA extract was obtained by combining 0.8 ml sample and 0.4 ml trichloro acetic acid (TCA); afterwards, the samples were put on ice for 10 min and centrifuged for 15 min at 6000 rpm. For TBARS determination in coronary venous effluent, the Krebs–Henseleit solvent was used as a blank control, while in the case of plasma sample distilled water was used [15].

#### 2.7.5. Determination of Antioxidant Enzymes (CAT, SOD)

Isolated RBCs were washed three times with three volumes of ice-cold 0.9 mmol/l NaCl, and hemolysates containing about 50 g Hb/l [16] were used for the determination of CAT activity [17]. Moreover, hearts from all animals were frozen at -80°C. Additionally, 0.5 section of each tissue was homogenized in 5 ml phosphate buffer (pH 7.4) on ice, and homogenates were centrifuged at 1200 x g for 20 min at 4°C. The resulting supernatants were used for the determination of cardiac activity of CAT and SOD. CAT buffer, prepared sample (hemolysates or heart tissue), and 10 mM H_2_O_2_ were used for CAT determination. Detection was performed at 360 nm. The amount of CAT was expressed as U/g tissue and in hemolysate as U/g Hb × 10^3^ [17, 18]. SOD activity was determined by the epinephrine method. Heart tissue or hemolysate was mixed with carbonate buffer, and then epinephrine was added. Detection was performed at 470 nm. The amount of SOD in heart tissue was expressed as U/g tissue and in hemolysate as U/g Hb × 10^3^ [19, 20].

#### 2.7.6. Determination of Reduced Glutathione (GSH)

The level of reduced glutathione (GSH) was determined based on GSH oxidation via 5,5-dithiobis-6,2-nitrobenzoic acid. GSH extract was obtained by combining 0.1 ml 0.1% EDTA, 400 *μ*l hemolysate, and 750 *μ*l precipitation solution (containing 1.67 g metaphosphoric acid, 0.2 g EDTA, 30 g NaCl, and filled with distilled water until 100 ml; the solution is stable for 3 weeks at +4C°). After mixing in the vortex machine and extraction on cold ice (15 min), it was centrifuged at 4000 rpm (10 min). Distilled water was used as a blank probe. The level of GSH was measured at 420 nm. The concentration is expressed as nanomoles per milliliter of red blood cells (RBCs) [21].

### 2.8. Histological Analysis

In order to evaluate the effects of *G. verum* on cell morphology after I/R, hematoxylin-eosin staining (HE) was performed for hearts according to the previously reported method [22].

### 2.9. Statistical Analysis

For statistical analysis of data within and between the control and *G. verum* group, IBM *SPSS* Statistics 20.0 for Windows was used. Three measured points were statistically analyzed: the first point was stabilization (S), the second was the first, and the last point of 30-minute reperfusion period (R1 and R7). Values were expressed as mean ± standard deviation (SD). Shapiro–Wilk test was used to check the distribution of data. Additionally, data was analyzed using a one-way analysis of variance (ANOVA) and the post hoc Bonferroni test for multiple comparisons. Values of *p* < 0.05 were considered to be statistically significant.

## 3. Results

### 3.1. Effects of *G. verum* Extract on Morphological Variables

Values of body weight (BW), heart weight (HW), and HW/BW ratio in both groups of rats are shown in Table 1. The HW/BW ratio was significantly lower in the *G. verum* group.

### 3.2. Effects of *G. verum* Extract on *In Vivo* Cardiac Function

LVIDd, LVIDs, and LVPWd were significantly decreased after *G. verum* treatment, while IVSd remained unchanged. Furthermore, midwall fractional shortening was significantly increased in the *G. verum* group in comparison to control (Table 2).

### 3.3. Effects of *G. verum* Extract on Ex Vivo Cardiac Function

**Figure 1:**
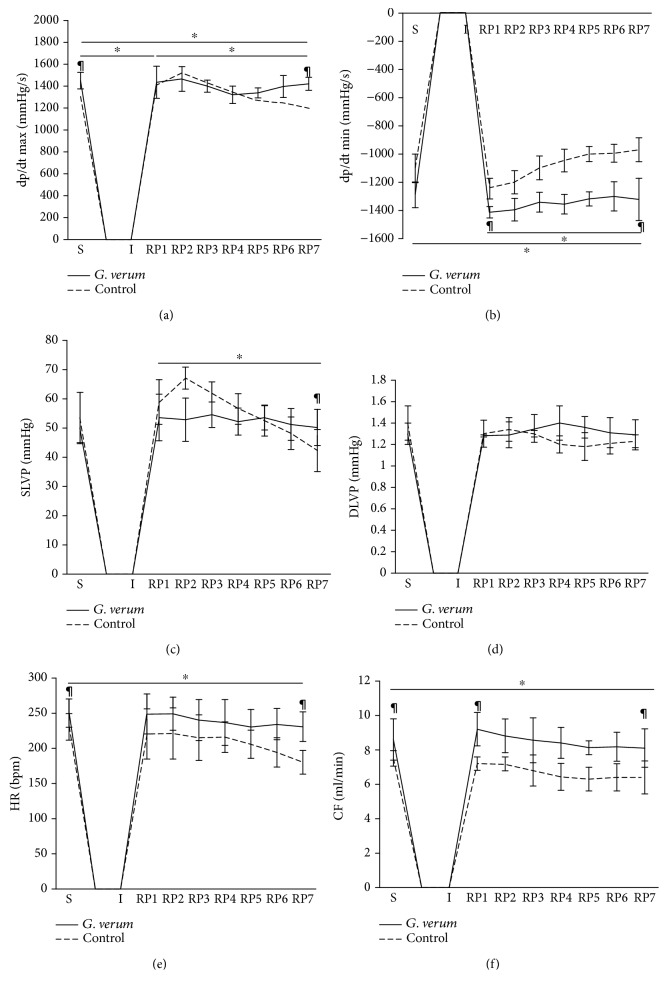
Effects of *G. verum* pretreatment on ex vivo cardiac function. (a) Comparison within and between groups in the value of dp/dt max, (b) comparison within and between groups in the value of dp/dt min, (c) comparison within and between groups in the value of SLVP, (d) comparison within and between groups in the value of DLVP, (e) comparison within and between groups in the value of heart rate, and (f) comparison within and between groups in the value of coronary flow. ^∗^Statistical significance at the level of *p* < 0.05 within the control group; ^#^statistical significance at the level of *p* < 0.05 within the *G. verum* group; ^¶^statistical significance at the level of *p* < 0.05 between the control and *G. verum* group. Data are presented as means ± SD. R1, first minute of reperfusion. R7, last minute of reperfusion.

There was a statistically significant increase in the value of the maximum rate of left ventricular pressure development (dp/dt max) at RP1 in comparison to S and RP7 in the control group and a decrease in RP7 in comparison to S point. In the *G. verum* group, the value of this parameter remained constant during the observed period. Furthermore, the values noticed in S and RP7 points were higher in *G. verum* than in the control group (Figure 1(a)). There was a drop in the value of the minimum rate of left ventricular pressure development (dp/dt min) at RP7 in comparison to RP1 and S points in the control group. Markedly, higher value of this parameter was noticed in the *G. verum* group both at RP1 and at RP7 compared to control conditions (Figure 1(b)).

A significant drop in systolic blood pressure in the left ventricle (SLVP) was found in the control group at RP7 when compared to RP1, while in the *G. verum* group SLVP value did not vary significantly. In the *G. verum* extract pretreated group, higher SLVP was observed at RP7 (Figure 1(c)). There was no statistically significant change in the value of diastolic blood pressure in the left ventricle (DLVP) both within and between groups (Figure 1(d)).

In the control group, a drop in heart rate (HR) was noticed at RP7 in comparison to S point. When compared to *G. verum* and control group, higher HR was noticed at RP7 and S points (Figure 1(e)). Coronary flow (CF) decreased at RP7 compared to S in the control group, and the value of the observed parameter was lower in this group in all points in comparison to the *G. verum* group (Figure 1(f)).

### 3.4. Effects of *G. verum* Extract on Systemic Redox State

**Figure 2:**
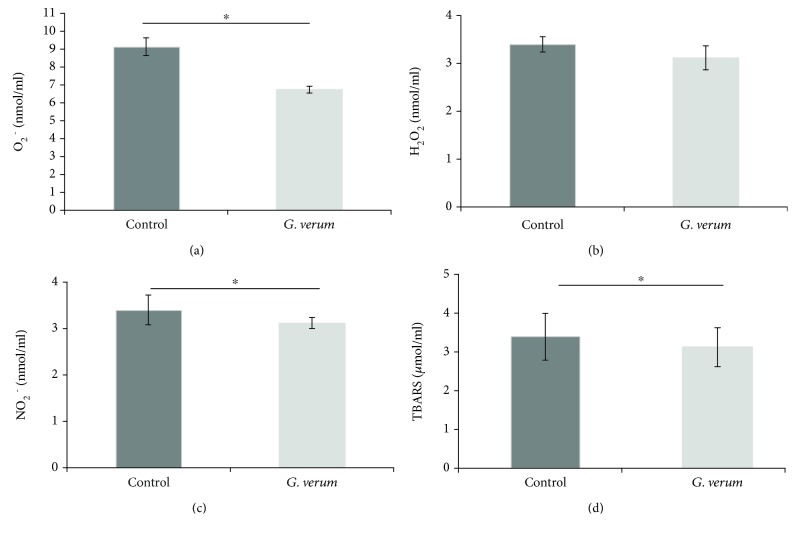
Effects of *G. verum* pretreatment on the level of prooxidants determined in plasma samples. (a) Comparison between groups in the value of O_2_^−^, (b) comparison between groups in the value of H_2_O_2_, (c) comparison between groups in the value of NO_2_^−^, and (d) comparison between groups in the value of TBARS. ^∗^Statistical significance at the level of *p* < 0.05 between the control and *G. verum* group. Data are presented as means ± SE.

**Figure 3:**
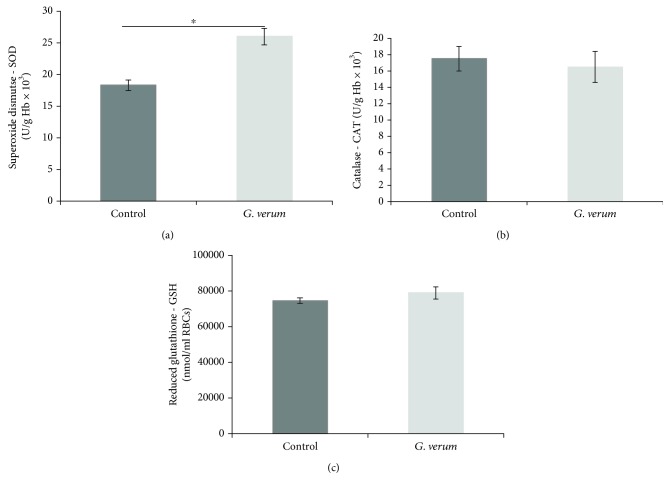
Effects of *G. verum* pretreatment on parameters of antioxidant defense system determined in erythrocytes samples. (a) Comparison between groups in the activity of SOD, (b) comparison between groups in the activity of CAT, and (c) comparison between groups in the level of GSH. ^∗^Statistical significance at the level of *p* < 0.05 between the control and *G. verum* group. Data are presented as means ± SE.

The levels of oxidative stress markers, such as O_2_^−^, NO_2_^−^, and TBARS, were decreased in the *G. verum* group in comparison to the control group, while there was no difference in the level of H_2_O_2_ (Figure 2). Regarding the parameters of antioxidant defense system, there was an increase in SOD activity in the *G. verum* group, while the activity of CAT and level of GSH were not changed compared to the control group (Figure 3).

### 3.5. Effects of *G. verum* Extract on Cardiac Redox State

#### 3.5.1. Superoxide Anion Radical (O_2_^−^)

**Figure 4:**
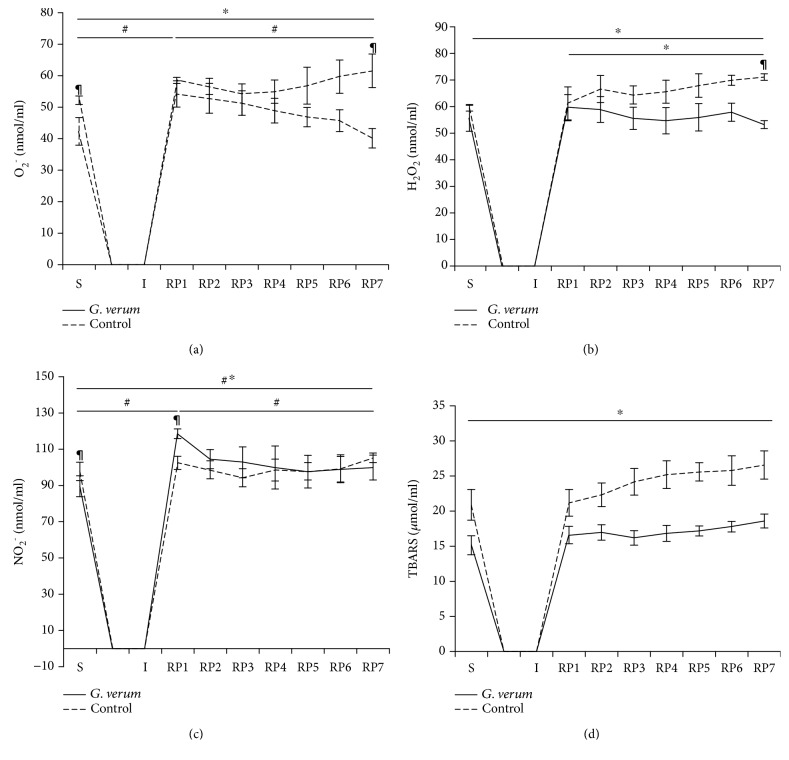
Effects of *G. verum* pretreatment on the level of cardiac prooxidants. (a) Comparison within and between groups in the value of O_2_^−^, (b) comparison within and between groups in the value of H_2_O_2_, (c) comparison within and between groups in the value of NO_2_^−^, and (d) comparison within and between groups in the value of TBARS. ^∗^Statistical significance at the level of *p* < 0.05 within the control group; ^#^statistical significance at the level of *p* < 0.05 within the *G. verum* group; ^¶^statistical significance at the level of *p* < 0.05 between the control and *G. verum* group. Data are presented as means ± SE. R1, first minute of reperfusion. R7, last minute of reperfusion.

There was a rise in O_2_^−^ at RP7 in comparison to S point in the control group. Furthermore, *G. verum* extract led to an increase in the level of this parameter in RP1 compared to S; however, it significantly decreased at RP7 and reached the values similar from the S point. The levels of O_2_^−^ both at S and RP7 moments were significantly higher in the control group (Figure 4(a)).

#### 3.5.2. Hydrogen Peroxide (H_2_O_2_)

A significantly elevated level of H_2_O_2_ was noticed in RP7 compared to S and RP1 in the control group, while in the *G. verum* group this parameter did not change during the time. A pronounced rise in the level of this oxidative stress marker was detected in the control group in comparison to *G. verum* at RP7 (Figure 4(b)).

#### 3.5.3. Nitrites (NO_2_^−^)

In the control group, there was an increase in the level of NO_2_^−^ in RP7 in comparison to S point. On the other hand, in the *G. verum* group an increase in the level of this parameter was noticed in RP1 compared to S and RP7 and in RP7 compared to the S point. In S and RP1 moments, the level of NO_2_^−^ was higher in the group pretreated with *G. verum* extract (Figure 4(c)).

#### 3.5.4. Index of Lipid Peroxidation Measured as TBARS

The level of TBARS was elevated in RP7 compared to S in the control group. Additionally, the level of this marker in the *G. verum* group in RP7 was significantly lower than in the control group (Figure 4(d)).

#### 3.5.5. Catalase (CAT) and Superoxide Dismutase (SOD)

**Figure 5:**
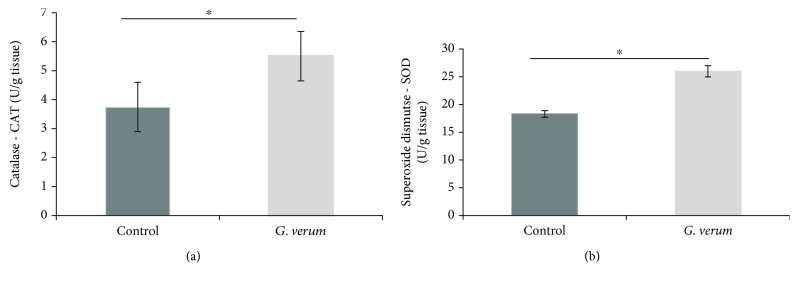
Effects of *G. verum* pretreatment on cardiac antioxidant enzymes activity. (a) Comparison between groups in the activity of CAT and (b) comparison between groups in the activity of SOD. ^∗^Statistical significance at the level of *p* < 0.05 between the control and *G. verum* group. Data are presented as means ± SE.

The cardiac activity of CAT and SOD was significantly increased in the *G. verum* group (Figure 5).

### 3.6. Effects of *G. verum* Extract on Heart Morphology

**Figure 6:**
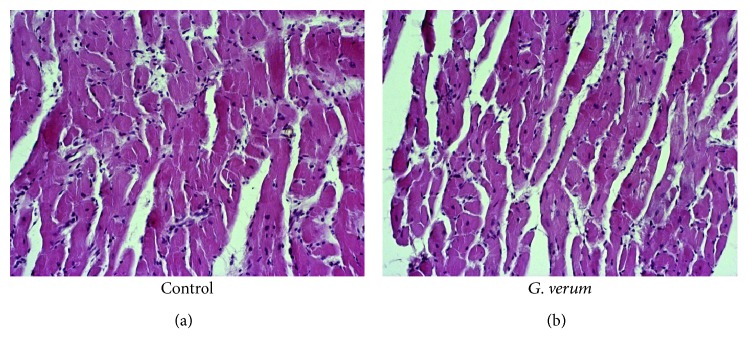
Histopathological changes in the myocardium following reperfusion *(magnification*, *×200*). Control group: hypertrophy of the cardiac muscle fibers, edema, necrosis, and zonal necrosis of higher number of cardiomyocytes, with hypereosinophilia, fragmentation of the fibers, and loss of nucleus. *G. verum* group: hypertrophy of the cardiac muscle fibers, edema, necrosis, focal necrosis of lower number of cardiomyocytes or unicellular, and less prominent degenerative changes.

Hypertrophy of the cardiac muscle fibers, edema, necrosis, and degenerative changes were observed in both groups. However, in the control group, degenerative changes were more prominent involving zonal necrosis of higher number of cardiomyocytes, with hypereosinophilia, fragmentation of the fibers, and loss of nucleus. Moreover, focal necrosis of lower number of cardiomyocytes or unicellular was found in the *G. verum* group (Figure 6).

## 4. Discussion

It is well established that hypertension may lead to left ventricular hypertrophy development, thus increasing mortality from AMI and vulnerability of myocardium to I/R injury [23, 24]. Advances in understanding the complexity of mechanisms involved in the pathogenesis of myocardial I/R injury points to a central role of oxidative stress. Therefore, growing scientific attention was focused on the modulation of redox homeostasis with plants and plant-derived products rich in natural antioxidants, thus reducing postischemic myocardial dysfunction [6]. Previous investigations have proven the antioxidant activity of *G. verum* extract *in vitro* [8, 25]. However, to our best knowledge, there is no data regarding its effects on heart function and potential of *G. verum* extract to attenuate myocardial damage, and diminish oxidative stress is open to discussion. We hypothesized that *G. verum* extract treatment might help salvage the ischemic-reperfused heart.

An echocardiographic examination of heart function demonstrated that 4-week *G. verum* treatment decreased LVIDd, LVIDs, and LVPWd and increased FS compared to the untreated SHR group, thus mitigating left ventricular hypertrophy and considerably improving cardiac function. This is in line with previous findings showing that natural substances belonging to a group of flavonoids might ameliorate endothelial dysfunction and cardiac hypertrophy in the spontaneously hypertensive rats [26, 27]. Additionally, the beneficial effect of isoflavones on overall cardiovascular markers in models of LV dysfunction, involving enhancement of percent FS, has been proven so far [28].

Our results clearly show that 20-minute ex vivo ischemia on *Langendorff* apparatus impaired myocardial function manifested as a marked reduction in dp/dt max, dp/dt min, SLVP, HR, and CF in the control group. However, in the *G. verum* group, values of dp/dt max and dp/dt min were unchanged during the reperfusion period, enabling the cardiac muscle to contract and relax without variations in force. In support of this finding, it was found that protective effects on ventricular contractility may be reached by either application of flavonoids alone or plant extracts containing polyphenolics, flavonoids, iridoids, and terpenoids [29, 30]. Moreover, in animals receiving *G. verum*, systolic function was not only preserved but even improved, as evidenced by an increase in SLVP. *G. verum* extract consumption prevented I/R-induced decline in HR, so unaltered HR provided enough time for the heart to contract strongly. Additionally, prolonged ischemia led to a coronary vascular dysfunction reflected in reduced CF in the control group. In the *G. verum* extract pretreated group, coronary flow was slightly but not significantly changing over 30 min recovery period, so at the end of reperfusion it returned to baseline values. This fact suggested that preconditioning with *G. verum* preserved the coronary vasodilatory response to I/R.

The presence of flavonoids in *G. verum* extracts was confirmed in previous studies [8, 9]. Striking evidence indicate that flavonoids have the potential to improve postischemic heart functionality [29, 30]. Polyphenols might inhibit endothelial NADPH oxidase (NOX), leading to a decrease in O_2_^−^ release and formation of peroxynitrite, thus preventing endothelial dysfunction [31]. Moreover, these compounds may activate endothelial nitric oxide synthase (eNOS), resulting in increase bioavailability of nitrogen monoxide (NO), the most potent endogenous vasodilator [32]. Therefore, we may speculate that the protective effects of *G. verum* extract on the maintenance of coronary vasodilator tone and coronary circulation may be attributed to the presence of total phenols.

Furthermore, we evaluated whether 4-week treatment with *G. verum* affects systemic redox homeostasis and alters systemic production of prooxidants, as well as the capacity of the antioxidant defense system. Our results showed that *G. verum* extract consumption exerted benefits referring to systemic redox state, leading to a drop in the plasma levels of O_2_^−^, NO_2_^−^, and TBARS. Moreover, we observed an increase in the activity of SOD, as the first line of cellular defense against oxidative injury, which is in line with a decrease in O_2_^−^ level. In fact, we may hypothesize that probably O_2_^−^ formed in mitochondria was converted to H_2_O_2_ in a reaction catalyzed by SOD. Our observation that there was no difference in the level of plasma H_2_O_2_ between groups is in accordance with unchanged CAT activity since the rise in H_2_O_2_ level would have led to a higher activity of CAT [33]. It has been proposed that polyphenols may preserve mitochondrial function and modulate redox signalling via inhibition of enzymes involved in ROS production such as xanthine oxidase (XO) and nicotinamide adenine dinucleotide phosphate-oxidase (NOX). Another way to exert antioxidant effect is via chelation of iron ions which catalyze several free radical-generating reactions [7].

In our research, I/R led to a rise in the generation of all cardiac prooxidants, which is consistent with the previous reports that during reperfusion of ischemic tissue the reversal of oxygen to oxygen-starved myocardium is associated with a burst of ROS generation. The main sources of prooxidants involve mitochondrial respiratory electron transport chain and activation of XO, resulting in enhanced production of very reactive species such as O_2_^−^ and H_2_O_2_ [34]. Nevertheless, pretreatment with *G. verum* extract was capable of preventing an increase in the levels of most of the markers of myocardial oxidative damage. Markedly, higher levels of TBARS, O_2_^−^, and H_2_O_2_ in coronary venous effluent in the control group compared to the *G. verum* group were noticed after 30 min restoration of blood flow, thus suggesting that *G. verum* extract attenuated oxidative stress derived from the endocardium of the left ventricle and endothelium of the coronary circulation. Increased activity of myocardial antioxidant enzymes supports a decline in cardiac production of prooxidants. In fact, enhanced SOD activity might be responsible for a decrease in O_2_^−^ levels, while lower level of H_2_O_2_ may be explained by its decomposition to water and oxygen catalyzed by CAT. In our study, *G. verum* treatment was associated with enhanced both systemic and myocardial SOD activity and diminished systemic and cardiac production of O_2_^−^, thus indicating that initial modulation of this antioxidant enzyme is involved in the action of *G. verum*. Consistent with these results, striking evidence indicate that polyphenols are able to activate endogenous antioxidant defense system, particularly SOD and CAT, thus alleviating oxidative stress-induced myocardial damage. In that sense, we may assume that this is one of the mechanisms through which polyphenol-containing extracts such as *Galium verum* extract may exert its antioxidant effects. Additionally, antioxidant potential of these natural compounds involves their ability to act as direct free radical scavengers as well [35, 36]. In addition to the aforementioned mechanisms, it has been known that flavonoids might alleviate I/R-induced oxidative stress by inhibiting the xanthine dehydrogenase/xanthine oxidase system [37]. Promising potential of *G. verum* extract in a decrease of oxidative damage may be mostly attributed to the additive and synergistic effects of bioactive molecules belonging to polyphenols.

Histological assessment suggested that ischemia altered the myocardial structure in both groups manifested as hypertrophia of cardiac muscle fibers, edema, necrosis, and degenerative changes. However, *G. verum* consumption alleviated I/R-induced deleterious effects on heart morphology and confirmed its potential to preserve both myocardial function and structure under ischemic conditions.

Our research highlighted for the first time that methanol extract of *G. verum* improved *in vivo* cardiac function and exerted benefits on systemic redox homeostasis. Additionally, it preserved the functional and morphological properties of the heart and prevented coronary vascular dysfunction after ischemia. Moreover, *G. verum* modulated the cardiac generation of prooxidants, thus alleviating oxidative stress-induced heart damage. Obvious protective effects of this plant species on myocardial I/R injury in our study provide a scientific basis for its use in triggering cardioprotection. However, further studies are certainly necessary for better understanding the underlying mechanisms.

## Figures and Tables

**Table 1:** Effects of *G. verum* extract on body and heart weight measurements.

	BW (g)	HW (mg)	HW/BW ratio
Control	266.65 ± 14.75	1.08 ± 0.07	4.95 ± 0.25
*G. verum*	235.75 ± 17.78	1.02 ± 0.1	4.32 ± 0.83^∗^

Values are shown as means ± SD. ^∗^Statistical significance at the level of *p* < 0.05 between the control and *G. verum* group; BW = body weight; HW = heart weight; HW/BW = heart weight to body weight.

**Table 2:** Effects of *G. verum* extract on *in vivo* cardiac function.

	Control	*G. verum*
IVSd (cm)	0.203 ± 0.011	0.208 ± 0.016
LVIDd (cm)	0.709 ± 0.003	0.458 ± 0.013^∗^
LVPWd (cm)	0.251 ± 0.013	0.176 ± 0.046^∗^
LVIDs (cm)	0.432 ± 0.01	0.186 ± 0.034^∗^
LVPWs (cm)	0.302 ± 0.002	0.297 ± 0.03
FS (%)	39.0 ± 2.34	59.3 ± 3.54^∗^

Values are shown as means ± SD. ^∗^Statistical significance at the level of *p* < 0.05 between the control and *G. verum* group; IVSd = end-diastolic interventricular septal thickness; LVIDd = left ventricular internal diameter end diastole; LVPWd = left ventricular end-diastolic posterior wall thickness; LVIDs = left ventricular internal diameter end systole; LVPWs = left ventricular posterior wall thickness; FS = fractional shortening.

## Data Availability

The data used to support the findings of this study are available from the corresponding author upon request.
